# Identifying promising or priority effective adolescent, sexual and reproductive health interventions in Ghana: what frameworks should guide the selection of interventions?

**DOI:** 10.1186/s12978-025-01989-0

**Published:** 2025-05-31

**Authors:** Daniel Malik Achala, Ama Pokuaa Fenny, Chris Atim, John Ele-Ojo Ataguba

**Affiliations:** 1 African Health Economics and Policy Association (AfHEA), Accra, Ghana; 2 Institute of Statistical, Social and Economic Research (ISSER), University of Ghana, Legon, Accra, Ghana; 3 Results for Development (R4D), Accra, Ghana; 4 Department of Community Health Sciences, Max Rady College of Medicine, Rady Faculty of Health Sciences, University of Manitoba, Winnipeg, MB Canada; 5 Partnership for Economic Policy, Duduville Campus, Kasarani, Nairobi, Kenya

## Abstract

**Background:**

Adolescent sexual and reproductive health (ASRH) is an integral part of the global health agenda. It is strongly featured in the universal health coverage (UHC) agenda of the sustainable development goals (SDGs). The need to expand ASRH services to accelerate progress on UHC is urgent in Africa, compared to other regions, given its youthful population and unmet ASRH needs. Limited access to ASRH services increases the risk and vulnerability of adolescents to poor health outcomes such as unintended pregnancies, high adolescent birth rate, poor birth outcomes, high maternal and neonatal mortalities and high exposure to sexually transmitted infections. The unavailability and inaccessibility of ASRH interventions to adolescents and young adults in most African countries, including Ghana, arise from several limitations, including inadequate funding of interventions, cultural barriers and norms, lack of education, and inadequate supplies of ASRH services and commodities, among others. However, gains from investments in ASRH interventions, especially following the implementation of the Millennium Development Goals, highlight the importance of identifying and prioritising adequate funding for effective ASRH interventions. This paper identifies priority ASRH interventions that can potentially advance the sexual and reproductive health (SRH) needs of adolescents in Ghana to accelerate progress towards UHC.

**Methods:**

Qualitative descriptive methods, combining literature review and stakeholder engagement, were used for this study. A literature review complemented by stakeholder engagement ensured the listing, ranking and validation of interventions.

**Results:**

Adapting an established framework designed by the West African Health Organization (WAHO) through stakeholders’ engagement process, the paper identifies four of seven priority interventions ranked and validated by stakeholders for addressing the SRH needs of adolescents in Ghana. Consistent with the literature, several interventions exist to address ASRH needs. The most effective priority or promising four interventions in Ghana, according to stakeholders, include adolescent health clubs programmes, girls’ empowerment programmes through comprehensive sexuality education, national capacity-building programmes to deliver high-quality integrated family planning and comprehensive maternal health services, and electronic health (eHealth)/digital health programmes.

**Conclusion:**

Identifying effective priority interventions for addressing the SRH needs of adolescents is a consultative process facilitated by proven and valid frameworks adapted to align with specific country contexts.

## Résumé

**Contexte:**

La santé sexuelle et reproductive des adolescents et jeunes (SSRAJ) fait partie intégrante du programme de santé mondial. Il figure en bonne place dans le programme de couverture sanitaire universelle (CSU) des objectifs de développement durable (ODD). En Afrique, la nécessité d’élargir les services de SSRAJ pour accélérer les progrès vers la couverture sanitaire universelle est urgente, par rapport à d’autres régions, compte tenu de la jeunesse de sa population et de ses besoins non satisfaits en matière de SSRAJ. L’accès limité aux services de SSRAJ augmente le risque et la vulnérabilité des adolescentes à des problèmes de santé tels que les grossesses non désirées, le taux élevé de natalité chez les adolescentes, les problèmes à la naissance, la mortalité maternelle et néonatale élevée et la forte exposition aux infections sexuellement transmissibles. L’indisponibilité et l’inaccessibilité des interventions de SSRAJ pour les adolescent(e)s et les jeunes adultes dans la plupart des pays africains, y compris au Ghana, découlent de plusieurs contraintes, notamment le financement inadéquat des interventions, les barrières et normes culturelles, le manque d’éducation et l’approvisionnement inadéquat en services et produits de SSRAJ, entre autres. Toutefois, les gains découlant des investissements dans les interventions de SSRAJ, en particulier après la mise en oeuvre des Objectifs du Millénaire pour le développement, soulignent l’importance d’identifier et de prioriser un financement adéquat pour des interventions de SSRAJ efficaces. Cet article identifie les interventions prioritaires en matière de SSRAJ au Ghana qui pourraient éventuellement faire progresser les besoins en matière de santé sexuelle et reproductive (SSR) des adolescents au Ghana afin d’accélérer les progrès vers la CSU.

**Méthodes:**

Des méthodes descriptives qualitatives, combinant une revue de la littérature et l’engagement des parties prenantes, ont été utilisées pour cette étude. Une revue de la littérature complétée par la mobilisation des parties prenantes a permis d’établir le recensement, le classement et la validation des interventions.

**Résultats:**

En adaptant un cadre établi conçu par l’Organisation ouest-africaine de la santé (OOAS) à travers le processus d’engagement des parties prenantes, l’article identifie quatre des sept interventions prioritaires classées et validées par les parties prenantes pour répondre aux besoins en matière de SSR des adolescents au Ghana. Conformément à la littérature, il existe plusieurs interventions pouvant répondre aux besoins en matière de SSRJA. Les quatre interventions prioritaires les plus efficaces ou prometteuses au Ghana, selon les parties prenantes, comprennent les programmes de clubs de santé pour adolescent(e)s, les programmes d’autonomisation des filles par le biais d’une éducation complète à la sexualité, les programmes nationaux de renforcement des capacités pour fournir des services de planification familiale intégrée de haute qualité et des services complets de santé maternelle, et les programmes de santé électronique (eHealth)/santé numérique.

**Conclusion:**

L’identification des interventions prioritaires efficaces pour répondre aux besoins des adolescent(e)s en matière de SSR est un processus consultatif facilité par des cadres éprouvés et valides adaptés à des contextes nationaux spécifiques.

## Background

Universal access to sexual and reproductive health (SRH) services is a fundamental goal within the Sustainable Development Goals (SDGs), particularly under Goal 3. Specifically, SDG target 3.7 aims to “ensure universal access to sexual and reproductive health-care services, including for family planning, information and education, and the integration of reproductive health into national strategies and programmes” by 2030 [1]. The global adolescent population, comprising approximately 16% of the world’s population, has generated a substantial demand for SRH services due to the pronounced SRH needs during adolescence [2]. Consequently, there is a critical need to enhance efforts to deliver quality SRH services. According to United Nations agencies, such as the World Health Organization (WHO), United Nations Children’s Fund (UNICEF), and United Nations Population Fund (UNFPA), adolescents are defined as individuals aged 10 to 19 years. Those aged 10 to 24 years are categorized as adolescents and youth, while youth specifically refers to those between 15 and 24 years [3, 4]. This paper adheres to these definitions in its discussion.

The need for expanded and quality SRH services in sub-Saharan Africa (SSA) is particularly urgent, given that the region has the highest proportion of adolescents globally. Adolescents in SSA account for approximately 20% of the global adolescent population and represent about 23% of the sub-region's total population [5]. Within the Economic Community of West African States (ECOWAS), adolescents make up roughly 23% of the population, while young people aged 10–24 years constitute around 32% [5]. In Ghana, adolescents represent about 22% of the population, with an additional 9% falling between 20 and 24 years [5]. Overall, young people aged 10–24 years comprise approximately 30% of the Ghanaian population.

Despite the substantial demand for SRH services driven by the global adolescent and youth population, access to these services remains constrained, particularly in sub-Saharan Africa (SSA). Contraceptive access is notably challenging in SSA, which has the lowest level of contraception knowledge compared to any other region globally [6, 7]. This limited coverage is reflected in family planning data, with only 56.3% of family planning needs met using modern methods, compared to 75% to 77% in regions like Asia and Europe [8]. Additionally, data from the Demographic and Health Surveys (DHS) indicate that contraceptive use is below 20% in two-thirds of SSA countries, with West African countries showing particularly low levels, often under 5% [9].

The limited access to sexual and reproductive health (SRH) services and information, leading to unmet demand, imposes a significant burden on the region's adolescents and young population. Globally, pregnancy-related complications account for an average of 600,000 deaths annually, with low- and middle-income countries—predominantly in Africa—shouldering approximately 94% of this burden [10–12]. Adolescence, marked by transitional and often risky behaviors, can result in lifelong adverse consequences for health, education, and socio-economic prospects [13–16]. Notable outcomes include teenage pregnancies, maternal mortality, and increased exposure to sexually transmitted infections (STIs) like HIV/AIDS. In Africa, adolescent pregnancy prevalence is estimated at 18.8%, with one-third of unintended pregnancies occurring among adolescent girls [17, 18]. Early sexual debut, with one in four adolescents engaging in sexual activity before age 15, exacerbates these challenges [19]. Furthermore, less than half of African adolescents report using condoms during their last sexual encounter [9], and nearly one in five adolescent boys in Eastern and Southern Africa tested positive for HIV in the past year [20]. Pregnancy-related complications and HIV/AIDS are leading causes of adolescent mortality in sub-Saharan Africa [21]. Additionally, inadequate funding remains a critical challenge to improving SRH services, particularly in countries like Ghana [22, 23].

In Ghana, significant disparities exist between adolescent boys and girls in accessing sexual and reproductive health (SRH) services. Given the country's substantial adolescent population, the demand for SRH services, including contraception, is understandably high. However, this demand remains largely unmet due to various barriers, such as the unavailability of contraceptives, social stigma, and a lack of education. Approximately 51% of sexually active adolescent girls in Ghana, who wish to avoid or delay pregnancy, do not use any contraceptive method [24]. The unmet need is even more pronounced among sexually active girls who are unmarried, at about 68%, and is higher among adolescents in lower wealth quintiles [24]. Among married adolescents, 22% use modern contraceptives, compared to only 18% of unmarried sexually active adolescent girls [24]. Additionally, only 28% of adolescents report using a condom during their last sexual encounter with a non-marital or non-cohabiting partner, with 26% of girls and 34% of boys doing so [24]. Educational disparities further compound these issues, with only 68% of girls aged 15–19 years attending school compared to 76% of boys in the same age group [24]. These challenges in Adolescent Sexual and Reproductive Health (ASRH) are likely contributors to the widening gender gap, particularly regarding early pregnancy and childbirth [25].

Despite the challenges and vulnerabilities faced by young adults and adolescents in Ghana, there have been notable gains from sexual and reproductive health (SRH) interventions over the years. Following the implementation of the Millennium Development Goals (MDGs), significant investments in maternal health interventions have led to a reduction in maternal mortality, from 499 deaths per 100,000 live births in 2000 to 263 deaths per 100,000 live births in 2020. Additionally, the rate of teenage pregnancies, although still concerning, has decreased from 21.5% in 1993 to 16% in 2014 [26].

Given the persistent challenges in Adolescent Sexual and Reproductive Health (ASRH) and the demonstrated impact of the Millennium Development Goals (MDGs), prioritizing funding for high-impact and effective interventions is critical. Such prioritization necessitates a careful process of identifying, analyzing, and selecting proven or promising interventions that comprehensively address the diverse health needs of adolescents, including sexual and reproductive health (SRH), nutrition, sexually transmitted infections (STIs), mental health, tobacco use, and substance abuse. Effective SRH interventions are typically integrated, cross-cutting, and multifaceted, requiring the active contribution of diverse stakeholders at both policy and service delivery levels [27–29]. The key question, therefore, is how to effectively identify these priority or promising ASRH interventions.

This paper details the processes undertaken in Ghana to identify priority or promising Adolescent Sexual and Reproductive Health (ASRH) interventions, aiming to guide more targeted investments. These processes were developed in alignment with the framework established by the West African Health Organization (WAHO) and were supplemented by local stakeholder engagement. The WAHO framework emphasizes that priority interventions should be cross-cutting and multifaceted, reflecting the complexity and multidisciplinary nature of SRH [27, 28]. It provides a comprehensive guide for developing national strategies for ASRH. Although several approaches can be employed in selecting priority ASRH interventions, the WAHO framework advocates for a method that focuses on the determinants and strategic objectives of ASRH [28]. Consequently, the framework utilizes the determinants of ASRH to account for the strategic objectives of Economic Community of West African States (ECOWAS) countries. Given the varying levels of ASRH intervention development, the WAHO framework identifies a list of promising or priority ASRH interventions that ECOWAS countries, including Ghana, can adopt, tailored to their unique contexts and aligned with the health determinants of adolescents and youth [28]. The framework and its application in this context are discussed in detail in subsequent sections of this paper.

## Methods

This paper employs a descriptive qualitative analysis of priority Adolescent Sexual and Reproductive Health (ASRH) interventions in Ghana, complemented by extensive stakeholder consultations. Initially, a literature review was conducted to assess the state of ASRH in Ghana. This review involved compiling a list of national ASRH interventions implemented across various regions. The process included an extensive review of national ASRH policies, programs, interventions, and financing documents. A comprehensive Google search was performed to locate relevant articles, policy documents, and strategic plans using keywords such as “Adolescent Sexual and Reproductive Health Policy,” “Strategy,” “ASRH Ghana,” “SRH Ghana,” “Sexual and Reproductive Health Ghana,” and “Reproductive Health Ghana.” This search was further supplemented by an online review of key websites, including those of the Ministry of Health, Ghana Health Service (GHS), World Health Organization (WHO), United Nations Population Fund (UNFPA), United Nations Children's Fund (UNICEF), and other UN agencies. Additional documents were sourced from stakeholders during conferences and meetings, such as the inception and dissemination workshops. The broad Google search strategy was designed to capture both grey and published literature, including documents from international organizations and government sources that may not be indexed in major databases. The effectiveness of this literature review approach in identifying relevant interventions was validated using feedback from key stakeholders.

Secondly, given that the primary focus of this study was to identify and select promising or priority Adolescent Sexual and Reproductive Health (ASRH) interventions in Ghana, the study population comprised all organizations involved in the implementation and coordination of ASRH interventions and activities at the national level. A stakeholder mapping exercise was conducted to identify the relevant stakeholders for participation in the identification and validation of priority ASRH interventions. This stakeholder engagement process complemented the literature review by providing additional documents not previously captured, validating the interventions identified through the literature review, and collaboratively selecting and validating these interventions using the West African Health Organization (WAHO) framework as the guiding criteria. This process was essential to ensure the acceptability and prioritization of interventions for implementation by the stakeholders.

Key stakeholders engaged in the study were identified using a snowball approach. The final sample was a mix of public organisations, agencies and institutions, non-governmental organisations (NGOs), civil society organisations (CSOs), private sector organisations and development partners working in the ASRH space in Ghana. For data reliability, 20 key informants were purposely and conveniently selected from stakeholder institutions responsible for delivering ASRH-related interventions in Ghana at the national level, either directly or indirectly. Direct intervention provider institutions were those delivering ASRH services directly to adolescents, while indirect service providers involved those performing oversight responsibilities, research and advocacy, policymaking functions, and financing of ASRH interventions. The list of institutions is presented in Table 1.

**Table 1:** Stakeholders who participated in the priority interventions selection and validation workshops

No	Participating Institution
1	Ministry of Health (MoH)
2	Ministry of Gender, Children and Social Protection (MoGCSP)
3	Department Of Social Welfare—Ghana
4	Ministry of Education (MoE)
5	Ghana Health Service (GHS)
6	National Population Council (NPC)
7	United Nations Population Fund (UNFPA)
8	World Health Organization (WHO)
9	United Nations Children's Fund (UNICEF)
10	Marie Stopes International, Ghana (MSI)
11	Plan International, Ghana
12	West African Health organisation (WAHO)
13	National Health Insurance Authority (NHIA)
14	National Youth Authority (NYA)
15	Alliance for Reproductive Health Rights (ARHR)
16	University of Ghana, Institute of Statistical Social and Economic Research (ISSER)
17	Plan Parenthood Association Ghana (PPAG)
18	Population Council, Ghana
19	Curious Minds, Ghana
20	DKT—Ghana

Finally, stakeholder consultations and interviews were conducted to ensure that any interventions potentially missed during the literature review were identified. These consultations included online meetings, electronic communications, face-to-face engagements, and two workshops: a validation workshop and a dissemination workshop. The first phase of these consultations and interviews focused on identifying additional interventions that may not have been captured during the literature review. The second phase involved collaboratively selecting priority interventions using the WAHO criteria, validating the selected interventions, and disseminating the study's findings.

### Developing a framework for identifying priority interventions

An analytical framework was also developed based on the framework established by the WAHO. The framework contains five major elements, namely: interventions targeting the social determinants of ASRH; proximal social determinants; knowledge, behaviour and lifestyle; adolescents and youth health problems; and lastly, a crosscutting element. The first four elements were adopted from the WAHO framework, identifying them as key elements of promising interventions.

Reviews of the literature demonstrate that the WAHO framework for identifying promising ASRH interventions is both extensive and comprehensive, with interventions identified using this framework often yielding long-term social benefits. According to the WAHO framework, an intervention is considered promising for potential scale-up if it is multifaceted and crosscutting, extending across various sectors (multisectoral). The WAHO criteria outline key elements of a priority or promising intervention, including targeting the social determinants of health, proximal social determinants, the biological/physiological and pubertal transition needs of adolescents, and their psychological needs. Additionally, promising interventions should address knowledge, behaviour, and lifestyle, health problems, mortality, and should be aligned with the country's overall response.

In this analysis, we adapted the WAHO framework, focusing on five major elements of a promising intervention: targeting the social determinants of health for adolescents and youth; engaging parents/guardians and the community as key players (proximal social determinants); addressing knowledge, behaviour, and lifestyle; tackling adolescents' and youths' health problems; and aligning with the country’s response strategies (Table 2). While it is ideal for promising interventions to encompass all four elements as outlined in the WAHO criteria, stakeholders concurred that an intervention meeting more than one of these elements could still be considered promising. The WAHO framework emphasizes that the complexity and multidisciplinary nature of ASRH necessitate an integrated approach, combining strategies and interventions to maximize the likelihood of successful implementation and desired outcomes.

**Table 2:** Dimensions of promising interventions to address ASRH challenges. Source: Adapted from the West African Health Organisation (2016)

Major health determinants among adolescents and youths	Possible priority interventions
Targeting social determinants	• Education in general, especially secondary education • Promoting and strengthening legal or policy measures with the • Participation of adolescents and youth • Promoting the economic “empowerment” of girls • Wide dissemination of information through communities, schools, and traditional and new media
Targeting proximal social determinants	• Promoting the economic empowerment of girls • Promoting parental/guardian commitment and communication between parents and children • Peer education is adequately implemented • At the community level: o Mobilisation of adults and community leaders o Sensitisation of boys and men for the promotion of norms of equity and gender
Targeting knowledge, behavior and lifestyle	• Creation of safe environments for girls (establishing trust, promoting communication and dialogue) • Wide dissemination of information through communities, schools and the media • Adolescent and youth-friendly centres adequately established • Peer education implemented within programmes • Promoting parental/guardian commitment and communication • Promoting programmes targeting sexual partners • Development of mentoring programmes through champions, role models and advocates • At the community level: o Mobilisation of adults and community leaders o Sensitisation of boys and men for the promotion of norms of equity and gender o Combination of interventions, training of service providers, improvement of appropriate service facilities o Wide dissemination of information through communities, schools and traditional and new media, including social media o Development of standards of quality service to adolescents and youth (Manual proposed by WHO) o Access to contraception
Targeting adolescents and youth health problems	• Combination: o Training of service providers o Improving appropriate service facilities o Wide dissemination of information through communities, schools and traditional and new media • Establishment of adolescent and youth-friendly health centres • Development of standards of quality services for adolescents and youth • Access to contraception • Focused antenatal care for pregnant adolescents • Improved road safety standards (wearing of seat belts and helmets)
Targeting country response	• Quality of coordination • Efficacy of multidisciplinarity • Involvement of all stakeholders, including adolescents and young people • Existence of disaggregated data on adolescents and young people • Alignment of interventions on the needs and priorities of adolescents and young people
Cross-cutting factors	• Sustainability of interventions • Effective coordination • Alignment of interventions to the needs and priorities of adolescents and the country’s health objectives

Additionally, the authors considered the crosscutting element as a crucial component of the criteria for selecting priority interventions in Ghana. This consideration followed a comprehensive review and analysis of the WAHO criteria, further refined through stakeholder consultations and validation. Crosscutting issues in ASRH, including the sustainability of interventions, effective coordination, and the alignment of interventions with the needs and priorities of adolescents and the country’s health objectives, were deemed essential. Therefore, these aspects were incorporated into the framework to ensure a holistic approach to addressing ASRH challenges.

## Results

### Overview of ASRH interventions identified and selected

A comprehensive list of Adolescent Sexual and Reproductive Health (ASRH) interventions in Ghana was compiled following an extensive literature review, which included an analysis of national policy documents and strategic plans. This list was subsequently shared with ASRH stakeholders across Ghana for their review and input. Additionally, the proposed framework for selecting priority or promising ASRH interventions was circulated among a broader group of stakeholders for further feedback. To refine this process, a stakeholders' workshop was convened, where participants engaged in brainstorming sessions to discuss the identified interventions and validate the selection criteria. Through this collaborative effort, a consensus was reached on a final list of seven broad ASRH interventions, aligned with the validated WAHO framework criteria, to be prioritized in Ghana.

Seven broad Adolescent Sexual and Reproductive Health (ASRH) interventions were identified through a comprehensive literature review, as documented in various strategic policies [30–35]. The Ghana Health Service (GHS) spearheaded the needs assessment and priority setting for adolescent health and development, in collaboration with key stakeholders. Furthermore, an Adolescent Technical Working Group, comprising representatives from institutions such as the Ministry of Health, Ministry of Education, Ministry of Gender, Children and Social Protection (MoGCSP), GHS, UNFPA, UNICEF, civil society organizations (CSOs), and private sector entities, played a crucial role in supporting the harmonization and implementation of these interventions. These ASRH programs are implemented by key stakeholders, including government agencies, major international funding bodies, NGOs, CSOs, and private sector actors in Ghana.

#### Adolescent health clubs

In Ghana, adolescent health clubs provide a structured forum where adolescents can navigate the challenges related to Adolescent Sexual and Reproductive Health (ASRH). Through this model, trained mentors facilitate the sharing of ASRH experiences and offer guidance on best practices for sexual and reproductive health. These clubs foster a peer network that enables adolescents to exchange experiences and learn effective strategies for addressing ASRH challenges. The environment within these clubs is designed to be safe and supportive, allowing adolescents to build relationships, enhance self-esteem, and develop positive habits such as delaying sexual activity, using contraceptives, and initiating HIV testing.

The intervention includes both school-based health clubs, covering basic and secondary schools, and out-of-school health clubs. It complements sexual and reproductive health (SRH) education and information dissemination in both school and out-of-school settings, and supports service provision through school-based health centres (SBHC). The adolescent health club intervention in Ghana aligns with the dimensions of the adapted West African Health Organization (WAHO) framework for effective ASRH interventions. Peer education and mentorship address social determinants, while the engagement with communities, schools, faith-based organizations, and civil society organizations (CSOs) reflects the intervention’s focus on proximal social determinants. Additionally, the intervention's health promotion component targets knowledge and behavior changes, and the provision of youth-friendly health services aligns with WAHO’s criteria for addressing adolescent health problems.

#### Empowering adolescent girls through comprehensive sexuality education (CSE)

This intervention includes programs, services, and activities aimed at enhancing comprehensive sexuality education (CSE) and sexual and reproductive health (SRH) services for adolescent girls in Ghana. Examples include advocacy initiatives and school-based programs that educate adolescent girls on SRH topics. The intervention is crosscutting, addressing major dimensions of the West African Health Organization (WAHO) criteria. It focuses on improving adolescent girls' health, education, and well-being (social determinants) through a multisectoral approach. This approach involves collaboration with the government, civil society organizations (CSOs), traditional authorities, faith-based organizations, academia, the private sector, and the media at both national and decentralized levels in Ghana (proximal social determinants).

Mentorship, school health clubs, safe spaces, and social media activities are key entry points for empowering adolescent girls and enabling their active participation in promoting access to gender-responsive CSE and youth-friendly SRH services. These components of the intervention align with the knowledge and behavior change and adolescent health problems dimensions of the WAHO criteria.

#### Strengthening national capacity in delivering high-quality integrated family planning and comprehensive maternal health services

The intervention targets programmes that support national capacity in addressing SRH. Programmes and projects targeted under this intervention include education on contraceptive use, advocacy programmes, and the numerous projects financed by international organisations that align with the government’s strategic plans in effectively integrating family planning services into maternal health. Under this intervention, there are sixteen modular training programmes for service providers and managers to empower them to provide quality youth-friendly health services. The training content captures key adolescent health problems such as STIs, pregnancy, abortion, and its complications, as well as the management of other conditions. Capacity strengthening for SRH service providers and agencies to integrate SRH services into maternal health better and provide quality youth-friendly services is crosscutting and multisectoral, involving diverse stakeholders such as government agencies, donors, CSOs and private sector players, and this feature of the programme aligns with the proximal social determinants criteria of the WAHO framework. The intervention also targets improved access to family planning and contraceptives, which supports the elements of knowledge and behaviour and adolescent health problems components of the WAHO criteria.

#### Digital platforms to support advocacy

This intervention enables the smooth delivery of comprehensive education for adolescents to share knowledge and resources effectively. The platforms also provide mechanisms for linking users to SRH services in Ghana. The digital health intervention in Ghana focuses on two elements of the WAHO criteria in addition to being crosscutting as it is sustainable and less costly. It addresses knowledge and behaviour change and adolescent health problems. The programme uses social media to engage young people and disseminate health information, which influences adolescents’ knowledge and behaviour. In terms of adolescent health problems, the digital health programme developed some Apps that are used to provide online counselling services, such as the “You Must Know” (YMK) App, by trained service providers. The programme also developed the “BoaMe” App to enhance services provided to victims of sexual and gender-based violence (SGBV) in Ghana.

#### Safety net programme

This programme supports adolescent pregnant girls through counselling, linkages and referrals for social services. The programme supports pregnant adolescent girls in Ghana by providing counselling services, providing adolescent health education, supporting them to return to school and providing them with health services and nutrition.

#### Data to address inequalities and achieve the SDGs

This programme involves data collection on ASRH indicators to assess progress towards achieving national and SDG targets. It includes data for monitoring and evaluation purposes of ASRH programmes and reporting purposes. The Ministry of Health, GHS, the Ghana Statistical Service and other allied agencies routinely collect data on ASRH indicators. The data inform planning, policies and programmes to address ASRH challenges.

#### Nutrition programme—girls’ iron-folate tablet supplementation programme (GIFTS)

Data from the 2014 Demographic and Health Survey (DHS) indicated that approximately 48% of adolescent girls aged 10–13 years were anaemic. In response, the Girls’ Iron-Folate Tablet Supplementation (GIFTS) program was introduced nationally in 2019. This program provides iron-folate tablet supplementation to both in-school and out-of-school adolescent girls aged 10–19 years through schools, health facilities, and other distribution channels, aiming to reduce anaemia levels among adolescent girls across the country. The Ghana Health Service (GHS) leads the implementation in collaboration with the Ghana Education Service and development partners such as UNICEF.

The identified interventions were shared online through email with the selected stakeholders for the study. Stakeholders were asked to confirm the accuracy of these interventions based on the literature review and were given the opportunity to suggest additional interventions. Where email responses were not received, follow-up interviews were conducted via Zoom to gather further input. Following these stakeholder consultations, it was unanimously agreed that the identified interventions represent the core ASRH interventions in Ghana, all of which have widespread nationwide implementation. No additional interventions were identified.

### Ranking of selected promising ASRH interventions

A ranking process was conducted among 20 stakeholders from various institutions to determine the four most critical or promising ASRH interventions. During the intervention selection and validation workshop, participants were provided with hard copies of the WAHO criteria, which were used to guide their rankings. The research team offered additional explanations to ensure that all participants fully understood the criteria and applied them accurately during the selection process.

The voting process involved stakeholders from public, private, and non-governmental organizations. Participants ranked the seven interventions in order of importance, from the most promising (ranked 1) to the least promising (ranked 7), based on their broad alignment with the framework's dimensions. Votes were disclosed through a show of hands, with results recorded on a flip chart. The rankings from the sheets were also collected, collated, and displayed, with no discrepancies noted between the voting methods.

Adolescent health clubs emerged as the top priority ASRH intervention in Ghana, followed by empowerment programs for adolescent girls through comprehensive sexuality education. The third-ranked intervention was national capacity strengthening programs aimed at delivering high-quality integrated family planning and comprehensive maternal health services. The fourth priority was digital health programs (Table 3). The interventions are described in rank order, from the most to the least promising.

**Table 3:** Results of the ranking of ASRH interventions by stakeholders

Identified interventions	Number ranking as 1st intervention	Number ranking as 2nd intervention	Number ranking as 3rd intervention	Number ranking as 4th intervention	Number ranking as 5th intervention	Number ranking as 6th intervention	Number ranking as 7th intervention	Total score by 1st position ranking	Rank
Adolescent health clubs	19	0	1	0	0	0	0	19	1st by voting count & consensus
Safety net programme	3	0	1	3	4	2	6	3	5th by voting count & consensus
Nutrition Programme—girls’ iron-folate tablet supplementation programme (GIFTS)	0	0	2	1	0	7	10	0	7th by voting count & consensus
Strengthened national capacity in delivering high-quality integrated family planning and comprehensive maternal health services	10	2	3	3	0	2	0	10	3rd by voting count & consensus
Empowering adolescent girls through comprehensive sexuality education	13	3	2	1	1	0	0	13	2nd by voting count & consensus
Data to address inequalities and achieve SDGs	1	0	1	0	4	6	8	1	6th by voting count & consensus
E-Health (Digital platforms)	6	1	0	4	4	2	3	7	4th by voting count & consensus

Additionally, the project included an assessment of the cost of each intervention to inform policy and advocacy for increased funding allocation to these priority ASRH interventions. However, this paper primarily focuses on the processes used to identify these interventions. The identification process spanned from February 2020 to July 2021, with a stakeholder workshop held as the culminating event to validate the priority interventions. The study findings were continuously disseminated from July 2021 to August 2023.

## Discussion

Ghana’s Adolescent Health Service Policies and Strategies, notably the 2016–2020 and 2020–2025 strategy documents, along with the 2018–2022 Ministry of Gender, Children, and Social Protection (MoGCSP) strategy document, outline the priority health needs and challenges facing adolescents and youth [30–33, 36, 37]. These strategic documents provide the frameworks for implementing health services and other health-related interventions for adolescents and young people. In this discussion, we analyze these interventions using the WAHO framework, which guides the identification and selection of priority interventions.

Utilizing the WAHO criteria for identifying promising interventions, complemented by stakeholder engagement, four priority ASRH interventions were selected and ranked according to their alignment with the various elements of a priority intervention. The selected interventions were chosen based on their crosscutting nature, addressing multiple elements of a promising intervention as defined by the WAHO framework. Interventions addressing all five elements of the WAHO criteria were ranked higher than those aligning with fewer criteria.

Adolescent health clubs were ranked as the top priority ASRH intervention, as they were believed to address all five elements of a priority intervention. Stakeholders indicated that this intervention touches on the social determinants of adolescent health through education, mentorship, peer learning, and youth-friendly service provision. The intervention also addresses proximal social determinants, knowledge and behavior, lifestyle, and adolescent health problems through a multisectoral approach that involves engagement with schools, faith-based organizations, and CSOs [37]. This comprehensive approach ensures alignment with national policies on ASRH, Universal Health Coverage (UHC), and the Sustainable Development Goals (SDGs).

Empowering adolescent girls through Comprehensive Sexuality Education (CSE) was ranked second, addressing key components of a promising intervention, though less comprehensively than adolescent health clubs. This intervention focuses on adolescent girls' health, education, and well-being, reflecting the social determinants of a promising intervention. It employs a multisectoral approach involving government agencies, communities, CSOs, and traditional authorities, among others, to promote girls' access to CSE and youth-friendly SRH services. This comprehensive approach addresses proximal determinants, knowledge and behavior, lifestyle, and adolescent health problems, aligning with the WAHO framework.

The remaining two interventions, though multisectoral and crosscutting, were ranked lower than the first two and were perceived as less comprehensive in addressing the five dimensions of a promising intervention. These interventions primarily focused on the dissemination of information through various channels, including communities, schools, parents/guardians, and both traditional and new media. This underscores the importance of a multisectoral approach and the critical role stakeholders play in Adolescent Sexual and Reproductive Health (ASRH).

The identified 'priority' interventions are designed to address key behavioral and lifestyle changes, encouraging adolescents to adopt healthier lifestyles. For example, creating safe environments for both adolescent girls and boys through youth-friendly centers, such as adolescent health clubs and corners, offers sexual health education that positively influences their lifestyle and behavior. These programs also promote a safe environment for all. Additionally, the priority programs emphasize peer education to build trust, foster communication, and encourage dialogue. Some interventions, like adolescent health clubs and peer education, have integrated mentoring programs that utilize role models and advocates to improve access to ASRH information. The provision of quality youth-friendly services is a central component of these selected priority interventions.

Our selection of crosscutting, multisectoral, and multifaceted interventions as key strategies to significantly improve Sexual and Reproductive Health (SRH) outcomes is supported by empirical literature. A systematic review of Adolescent Sexual and Reproductive Health (ASRH) interventions that significantly impact SRH outcomes identified SRH education—particularly school-based, sports-based, counseling, and communication campaigns—as effective in positively influencing adolescents' and young people's SRH [38]. These crosscutting interventions were found to increase the use of modern contraceptive methods, reduce the number of sexual partners, promote safe sexual behaviors, decrease rates of sexually transmitted infections (STIs) and HIV/AIDS, and enhance the utilization of SRH services. These findings align with our stakeholder engagement process, which identified and prioritized adolescent girls' empowerment through comprehensive sexuality education and adolescent health clubs as high-impact interventions for addressing the SRH needs of adolescents in Ghana.

However, some authors question the effectiveness of digital-based interventions, suggesting that while they may improve knowledge, they may not necessarily lead to behavior change [38–40]. The authors argue that more randomized controlled research on digital interventions is necessary to adequately assess their effectiveness. Despite this, our engagement with stakeholders indicates that digital-based interventions are effective in addressing the SRH needs of adolescents by increasing their knowledge, which is likely to lead to safer SRH practices and behaviors.

Evidence indicates that comprehensive training for service providers and program managers in family planning, abortion, and counseling services effectively reduces unintended teenage pregnancies and unsafe abortions [38, 41]. Studies also highlight that comprehensive post-abortion family planning services are high-impact interventions; incorporating family planning into post-abortion care increases contraceptive use and reduces repeat abortions [41, 42]. These findings underscore the importance of prioritizing the scaling-up of "strengthened national capacity in delivering high-quality integrated family planning and comprehensive maternal health services" to address the SRH needs of adolescents in Ghana.

To achieve significant progress in ASRH indicators, the interventions identified in this research should guide nationwide scaling efforts, ensuring that their design aligns with local population needs and that they receive broad support from recipients. Given the gendered nature of ASRH issues, these interventions should prioritize young girls, who are often disproportionately affected by teenage pregnancies, unsafe abortions, and school dropouts. It is crucial to combine these promising or priority interventions to maximize their impact on addressing the needs of adolescent girls and boys in Ghana. Additionally, regular consultation with program recipients (adolescents) is recommended to assess the interventions' impact and to identify necessary modifications for enhanced effectiveness.

## Strengths and limitations

The paper's primary strength lies in integrating diverse approaches within an established framework to identify priority interventions. Additionally, the active involvement of key stakeholders in identifying promising and priority interventions further strengthens the process. However, the identification process is not without limitations. The search strategy, while comprehensive, may not capture an exhaustive list of interventions, even when supplemented by other strategies. Nonetheless, the engagement of key stakeholders in Ghana's ASRH landscape ensures that no significant interventions were overlooked.

Another limitation concerns the ranking methodology. Although using a weighted average of the scores could be an alternative, assigning appropriate weights poses a challenge. Moreover, the rankings in this paper prioritized interventions with higher scores, rendering the impact of a weighted average minimal. It is also important to note that participants' rankings were guided by specific criteria, but personal biases could have influenced their decisions. Additionally, the focus on nationally implemented interventions may have excluded promising interventions that are currently limited to a few districts or regions due to their limited geographic spread.

## Conclusions

Identifying priority interventions presents significant challenges; however, leveraging an established framework has simplified this process. The key interventions identified in this study were those endorsed by stakeholders as having a meaningful impact on Ghana’s broader health sector goals. The selection of these interventions as effective priorities is further validated by empirical literature, which demonstrates their positive impact on ASRH service uptake, knowledge, behavior change, and the overall health and well-being of adolescents.

In the context of Ghana, additional in-depth studies may be necessary to assess the conditions required for scaling these interventions to a wider target population. The success of these priority interventions will largely depend on their implementation and monitoring, including the resources allocated to them. A critical challenge remains the sustainability of funding, as many of these identified priority interventions are primarily supported by donor funds. The continuity of these interventions often hinges on the availability of consistent funding, particularly from government sources.

## Data Availability

The study did not involve any human subjects, and therefore no personal or sensitive data was collected. The data generated and analyzed during this study is available from the corresponding author on reasonable request.

## Abbreviations

AfHEA: The African Health Economics and Policy Association
AIDS: Acquired Immune Deficiency Syndrome
ASRH: Adolescent Sexual Reproductive Health
CSE: Complimentary Sexual Education
CSOs: Civil society organisations
DHS: Demographic and Health Survey
ECOWAS: Economic Community of West African States
GHS: Ghana Health Service
GIFTS: Girls’ iron-folate tablet supplementation programme
HIV: Human Immuno-deficiency Virus
MDGs: Millennium Development Goals
MoGCSP: Ministry of Gender, Children and Social Protection
MoH: Ministry of Health
NGO: Non-governmental organisations
SBHC: School-based health centres
SDGs: Sustainable Development Goals
SGBV: Sexual and gender-based violence
SRH: Sexual Reproductive Health
SSA: Sub-Saharan Africa
STIs: Sexually Transmitted Infections
UHC: Universal Health Coverage
UNFPA: United Nations Population Fund
UNICEF: United Nations Children’s Fund
WAHO: West African Health Organization
WHO: World Health Organization
YMK: You Must Know
